# Disrupted state transition learning as a computational marker of compulsivity

**DOI:** 10.1017/S0033291721003846

**Published:** 2023-04

**Authors:** Paul B. Sharp, Raymond J. Dolan, Eran Eldar

**Affiliations:** 1 Max Planck UCL Centre for Computational Psychiatry and Ageing Research, University College London, London, UK; 2 Wellcome Centre for Human Neuroimaging, University College London, London, UK; 3 The Hebrew University of Jerusalem, Jerusalem, IL, USA

**Keywords:** Anxiety, compulsivity, computational modelling, model-based learning, transdiagnostic

## Abstract

**Background:**

Disorders involving compulsivity, fear, and anxiety are linked to beliefs that the world is less predictable. We lack a mechanistic explanation for how such beliefs arise. Here, we test a hypothesis that in people with compulsivity, fear, and anxiety, learning a probabilistic mapping between actions and environmental states is compromised.

**Methods:**

In Study 1 (*n* = 174), we designed a novel online task that isolated state transition learning from other facets of learning and planning. To determine whether this impairment is due to learning that is too fast or too slow, we estimated state transition learning rates by fitting computational models to two independent datasets, which tested learning in environments in which state transitions were either stable (Study 2: *n* = 1413) or changing (Study 3: *n* = 192).

**Results:**

Study 1 established that individuals with higher levels of compulsivity are more likely to demonstrate an impairment in state transition learning. Preliminary evidence here linked this impairment to a common factor comprising compulsivity and fear. Studies 2 and 3 showed that compulsivity is associated with learning that is too fast when it should be slow (i.e. when state transition are stable) and too slow when it should be fast (i.e. when state transitions change).

**Conclusions:**

Together, these findings indicate that compulsivity is associated with a dysregulation of state transition learning, wherein the rate of learning is not well adapted to the task environment. Thus, dysregulated state transition learning might provide a key target for therapeutic intervention in compulsivity.

## Introduction

Although it is known that compulsivity, fear, and anxiety are associated with beliefs that external threats and internal worries are less controllable (Cartwright-Hatton & Wells, 1997; Grupe & Nitschke, 2013; Mathews, 1990), we lack a useful model to explain how such beliefs might develop. Psychological theorists propose that such beliefs stem from a lack of self-efficacy in controlling one's environment (Bandura, 1978), but this does not explain the nature of the dysfunction that produces a lack of self-efficacy. Here, we test a novel hypothesis that a deficit in state transition learning – specifically, the ability to internalize probabilistic maps of how actions lead to new states in the environment – is associated with fear, anxiety, and compulsivity symptoms (Zorowitz, Momennejad, & Daw, 2020).

An effort to relate these symptoms to state transition learning extends nascent work on how psychopathology is marked by difficulty in utilizing internal maps of one's environment (so-called ‘cognitive maps’) for decision-making (Behrens et al., 2018; Tolman & Honzik, 1930). Early computational psychiatry work has shown that ‘model-based’ behavioral control, which uses a cognitive map of state transitions to plan one's actions, is disrupted in individuals who show high compulsivity (Gillan et al., 2020a, 2020b, 2016; Rouhani et al., 2019). This work, however, has not considered that individuals may differ in how they learn state transitions. Consequently, model-based deficits were attributed to a failure to *use* state transition knowledge, the learning of which was assumed to be intact (Konovalov & Krajbich, 2020). Investigating state transition learning can thus determine whether compulsivity-related model-based deficits are due to a more fundamental impairment in learning how to transition between states. Doing so can additionally test for potential anxiety- or fear-related deficits in transition learning, which may be independent from the degree to which state transition knowledge is used for decision-making (e.g. Gillan et al., 2020b).

**Fig. 1.**
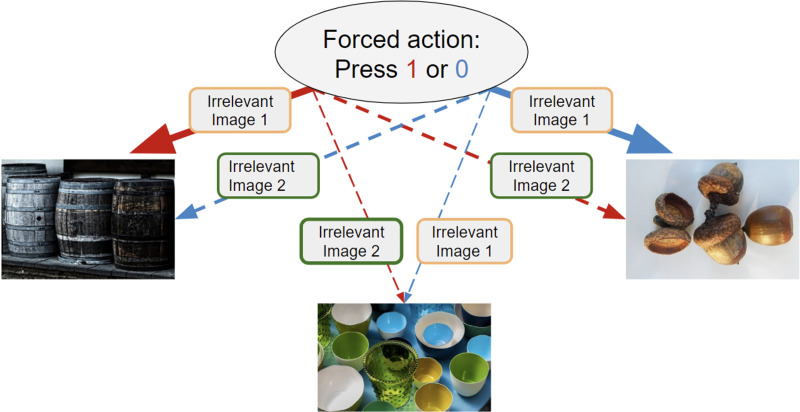
Study 1: one-step revaluation task learning phase. Participants played a one-step revaluation task where two actions could each lead to three possible states. Depicted here is the learning phase wherein participants were instructed to take an action (pressing either 1 or 0), and observe to which of three possible states (each denoted by an emotionally-neutral image) their action led. Each action led to each of the three images with different probabilities. The red arrows above symbolize transitions from action 1, and blue arrows from action 0. The thick arrow indicates the common transition, occurring 5/10 times (per action), the middle-thickness dashed arrow indicates the uncommon transition, occurring 3/10 times, and the thinnest dashed arrows the rarest transitions, occurring 2/10 times. Participants chose each action 10 times, and saw an ‘irrelevant’ image of either neutral or emotional valence presented before the outcome state. The instructions clarified these additional images were irrelevant and should be ignored. Irrelevant images were displayed for 500 msec. Analyses revealed a lack of a role of these irrelevant images on the relationship between psychopathology and state transition learning.

To address these issues, Study 1 tested whether difficulty in learning state transitions is associated with the symptoms of compulsivity, fear, and anxiety. For this purpose, we designed a task environment where it is difficult to learn state transitions, yet once learned it is trivial to use them for decision-making (Fig. 1). Moreover, unlike previous investigations, we specifically avoided administering rewards during the learning phase. This design enabled us to isolate state transition learning from other facets of learning and planning.

To investigate the relationship between transition learning and psychopathology, we sampled participants from the general population, and assessed them for a range of transdiagnostic symptoms, including those associated with depression, mania, worry, sympathetic hyperarousal, and compulsivity. Using this approach, we show that individuals high in compulsivity demonstrate impaired transition learning. In this regard, preliminary evidence implicated a transdiagnostic factor spanning compulsivity and fear.

Our novel one-step revaluation task is capable of isolating state transition learning from planning because it separates the learning and decision phases. However, for the same reason, it is not suited for modeling the computations underlying state transition learning. Thus, to more precisely characterize the computations involved in state transition learning, we complemented Study 1 with two independent studies comprising tasks which measured decision-making as state transition learning was ongoing. In Study 2, we re-analyzed publicly available two-step task data (Gillan, Kosinski, Whelan, Phelps, & Daw, 2016) to measure the rate of learning with regards to stable state transitions. In Study 3, we used a novel multi-goal pursuit task (Sharp, Russek, Huys, Dolan, & Eldar, 2021) to measure the rate of learning with regards to continuously changing state transitions. In both studies, the best-fitting model separately quantified the rate of state transition learning and the use of transition knowledge for making choices. Fitting the model to each individual participant revealed that compulsivity is associated with excessively fast transition learning with regards to stable transitions (which demand slower learning) and excessively slow transition learning with regards to changing state transitions (which demand faster learning).

Thus, across three independent studies, we demonstrate that compulsivity is associated with dysregulated state transition learning that fails to adapt to the statistics governing state transitions.

## Methods and materials

### Study 1: isolating state transition learning disruptions

#### Participants

We used the Gorilla Experiment Builder (www.gorilla.sc) to develop and host our experiment (Anwyl-Irvine, Massonnié, Flitton, Kirkham, & Evershed, 2020). Participants were recruited online through Prolific recruiting service (https://www.prolific.co/). The only inclusion criteria for the study were that participants spoke fluent English and were over 18 years old. The study (final sample = 174) comprised 119 male participants (68%). We recruited 219 participants based on a power analysis that provided 80% power to detect a small-medium effect size (*r* ⩽ 0.2), given similar effect sizes across a relevant prior literature examining links between psychopathology and assays of model-based learning (Alvares, Balleine, & Guastella, 2014; Gillan et al., 2016; Voon et al., 2015). Forty-five participants (20%) did not meet the criterion of accurately identifying the common image (to which an action led 80% of the time) in a practice round, and were thus excluded from further analysis. Participants gave written informed consent before taking part in the study, which was approved by the university's ethics review board.

#### Psychopathology assay

Before participants completed the online task, they filled out five questionnaires covering a range of transdiagnostic types of psychopathology. Worry was measured via the 16-item Penn State Worry Questionnaire (PSWQ; Meyer, Miller, Metzger, & Borkovec, 1990). Anxious arousal was measured with the 10-questions mini version of the Mood and Anxiety Symptom Questionnaire – Anxious Arousal subscale (MASQ; Casillas & Clark, 2000). Obsessive compulsiveness was measured with the Obsessive Compulsive Inventory – Revised (OCI-R; Foa et al., 2002). Depressive symptoms were measured with the Becks Depression Inventory – II (BDI; Beck, Steer, & Brown, 1996). Mania symptoms were measured with the Altman Self-Rating Mania Scale (ASRM; Altman, Hedeker, Peterson, & Davis, 1997; see online Supplementary Fig. S1 for full distributions of questionnaires).

Although IQ, age, and gender are known to covary with compulsivity and related forms of psychopathology, we chose not to measure them in the data we collected for two reasons. First, because we investigate a learning impairment, controlling for IQ, age, or gender might remove meaningful variance and diminish our ability to detect relations with psychopathology. Second, and most importantly, we do not question whether compulsivity is associated with known deficits in model-based control, as this association has already been demonstrated to exceed the effects of IQ, age, and gender (Gillan et al., 2016). Our main goal was to determine how state transition learning is related to compulsivity and whether it explains compulsivity-related deficits in the utilization of state transition knowledge for model-based control.

#### One-step revaluation task

Participants played a one-step revaluation task where two actions could each lead to three possible states. In a learning phase (Fig. 1), subjects were forced via instruction to take an action (pressing either 1 or 0), and observe to which of three possible states their action led. Each action led to each of the three images with different probabilities. Participants were told that the better they learned the states that commonly followed each action, the more money they would win, though unbeknownst to subjects the payment schedule for each round was fixed at £7. Participants chose each action 10 times, with the two actions randomly interleaved. Additionally, before the presentation of the outcome state, an irrelevant image of either neutral or emotional valence was presented on screen.

After participants completed the learning phase, participants faced a test phase in which they were presented with each of the relevant outcome-state images from the learning phase. Upon seeing the image, participants had to choose the action that delivered them to that image most often in the learning phase (e.g. choosing action 1 when shown the image of the barrels). No feedback was presented after a choice was made so that performance would solely reflect what was learned about transitions in the learning phase. Note that both actions always led to rare states with the same, low probability (20%), and thus there was no optimal action to take in the test phase for such queries (Fig. 1, transitions to cups image). We included the ‘rare state’ because we learned in piloting that this was necessary to remove a ceiling effect.

The experiment started with an easy practice round, where each action led to its ‘common’ image 16 out of 20 times (e.g. action 1 leading to the image of the barrels in Fig. 1), and twice to each of the other two images. Participants then played five blocks (henceforth, ‘experimental conditions’) each of which comprised a learning phase (Fig. 1; 20 forced choice trials) and a test phase (three queries). Four blocks differed in whether the irrelevant emotional stimuli were presented during learning along common or uncommon transitions and were of either positive or negative valence. The remaining block included only neutral stimuli.

Image stimuli were taken from the OASIS image dataset (Kurdi, Lozano, & Banaji, 2017). All task-relevant images were selected to have neutral emotional content (i.e. valence between 3.5 and 4.5 on a seven-point scale, and arousal was below 3.5). Irrelevant emotional images were selected with high arousal (>5) and valence (<3 for negative, >5 for positive).

Of note, the original intention of the task was to test whether the interaction between task-irrelevant affective images and pre-existing worry impacted state transition learning. Specifically, we hypothesized that chronic worry would produce an attentional bias to negative distracting images, and this would facilitate greater memory for such transitions. As such, we predicted those with chronic worry would perform better at test when negative distractors were paired with common transitions, and worse when negative images were paired rare transitions. However, contrary to this prediction, no effects were found for the pairing of negative images with common (mode = −0.39, CI −0.96 to 0.27) or rare transitions (mode = 11, CI −0.51 to 0.70; see online Supplementary Fig. S2).

#### Modeling whether state transition learning was impaired in the novel revaluation task

To quantify learning, we analyzed participants' choices from test queries for which there was an optimal action (acorns and barrels in Fig. 1). A Bayesian logistic regression model was used to predict whether the participant correctly learned the state transition matrix that characterized each block, as evidenced by their choices in the test phase. Specifically, the logistic regression predicts whether each participant choice was correct as a function of: a grand-mean intercept, a random-effect participant baseline, a random-effect experimental condition, and three fixed-effects for each transdiagnostic psychopathology factor. The intercept was drawn from a normal prior where the prior was centered on the grand mean (mean = 0.6, s.d. = 1). Each random-effect was drawn from a group-level prior whose mean was 0. We estimated hierarchically the group-level variances on each random effect. We kept all priors on variances across fixed- and random-effects to an upper bound of 3. The hyperpriors for the variance of the participant baseline were Uniform(0,3) whereas each fixed-effect for each transdiagnostic psychopathology factor was drawn from Normal(0,3). The participant-specific baseline and experimental condition effects were instantiated in STAN as unordered categorical variables.

To derive the transdiagnostic factors, we used the Factor Analyzer Python package (https://pypi.org/project/factor-analyzer/) with a promax rotation, which allows factors to be partially correlated, along with default parameters.

For hypothesis testing, we defined a Region Of Practical Equivalence (ROPE; Kruschke, 2018) representing the range of insignificant effect sizes, and compared it to the 95% most credible parameter values (i.e. the 95% Highest Density Intervals; HDI). The ROPE was defined as 10% of the standard deviation in the estimate of the baseline effect, signifying a small effect (0 ± 0.01).

Posterior probability distributions of effects were estimated using a Hamiltonian Monte Carlo (HMC) sampling procedure using a python implementation of STAN statistical software (Stan Development Team, 2020). We changed the adapt_delta parameter to 0.9 and max tree depth to 18, to reduce divergences and improve sampling efficiency.

After fitting each model, we re-scaled all categorical parameter estimates by enforcing a sum-to-zero constraint (i.e. the sum of coefficients for the different levels of the categorical variable equals 0). This procedure improves the interpretability of categorical variable coefficients and regression intercept by ensuring each coefficient represents the deviation for the corresponding categorical level from baseline, which is captured by the intercept (Kruschke & Liddell, 2018). In all models, all estimated parameters had good indicators of reliable sampling from the posterior, including r-hat below 1.1 and effective sample size above 1000 for all parameters.

### Study 2: quantifying disruptions in state transition learning when transitions are stable

To investigate how disrupted state transition learning in compulsivity manifests in model-based control when state transitions are stable, we fit computational models of state transition learning to a publicly available two-step task data (*n* = 1413; Gillan et al., 2016). Briefly, the two-step task is a trial-and-error learning task that requires participants first choose an action that leads them to a second state, from which they can choose another action in order to attain reward. Participants are instructed in training about the probability of common and rare transitions, which do not change over the course of the task, but participants are not told which of the two transitions for a given action is most likely and thus they must learn that through experience. We empirically tested here whether such learning is less effective in some individuals than in others, such that accounting for these individual differences would improve our ability to model participants' choices.

Previous models of this task learn state transitions by a simple counting heuristic (Otto, Raio, Chiang, Phelps, & Daw, 2013). We refer to this as the ‘Typical model’. Specifically, each trial, one of two possible transition matrices, is inferred as the true transition matrix that determines the probability first-stage actions transition second-stage states (e.g. either action 1 leads to state 1 70% of time, or to state 2 70% of the time). Here the columns are the possible two actions and the rows are possible states each action can transition to:1
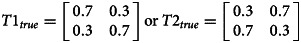


The transition matrix is inferred by using a running counter of how many times each action transitioned to each second stage state. For instance, if a subject's initial choice was action 1, and they transitioned to state 1, it would be encoded in the following ‘counting’ matrix:2
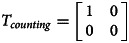


At any point, *T*1_*true*_is inferred if *T_counting_*(1,1) + *T_counting_* (2,2) >*T_counting_* (1, 2) + *T_counting_* (2, 1), where the indices in the parenthesis reflect (row,column) in *T_counting_*. If the inequality is the converse, *T*2_*true*_is inferred. If both sides of the inequality are equal, the average of *T*1_*true*_ and *T*2_*true*_ is inferred.

Here, we tested an alternative model that incrementally learns state transitions as a consequence of state transition prediction errors (Gläscher, Daw, Dayan, & O'Doherty, 2010). We refer to this model as the incremental state transition learning (ISTL) model (online Supplementary Fig. S3). This model quantifies each individual's rate of state transition learning, which when excessively low or high can result in inaccurate estimates of true state transition probabilities. For instance, if one transitions from action 1 to state 2 at time *t*, the estimated probability of that transition, *P*(*s* = 2|*a* = 1)*_t_*, is updated by the state prediction error [1–*P*(*s* = 2|*a* = 1)*_t_*] according to a learning rate, *γ*, which defines how much weight to give to the most recent prediction error:3
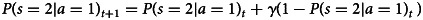


At the same time, the complementary transition probability, from action 1 to state 1, is also updated so that the probabilities sum to 1:4
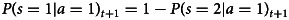


By contrast, estimated state transitions for the action that was not taken decay toward the initial prior of 0.5:5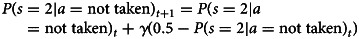 and similarly for *s* = 1.

Action values for chosen actions were updated exactly as in Gillan et al. (2016), with a decay rate on action values that equals the complement of the learning rate, 1-*α*. Action values for unchosen actions were also updated as in Gillan et al. (2016) except that they had their own decay rate, *D*.

We tested participants' choices against several additional models as detailed in online Supplementary Fig. S3. None of these models was able to explain choices as well as the ISTL model, and thus we focus here on a comparison between the Typical and ISTL models.

Models were fit hierarchically via iterative importance sampling (Bishop, 2006), which has been shown to ensure high parameter and model recoverability (Eldar & Niv, 2015; Eldar, Roth, Dayan, & Dolan, 2018). The priors for this model-fitting procedure largely do not affect the results, because the procedure iteratively updates priors via likelihood-weighted resampling in order to converge on the distributions of parameters that maximize model evidence. As such, all parameters had ‘naïve’ priors. To ensure models and parameters were appropriately designed, we simulated data using the ISTL and the Typical models, and successfully recovered both the true parameter values (all correlations between true and fitted values above 0.5; online Supplementary Fig. S4) and the model that generated each simulated dataset (online Supplementary Note 1).

### A model-agnostic behavioral signature of model-based behavior

To quantify individual differences in model-based control in empirical and simulated data without relying on a specific model, we used a behavioral signature established in prior work (Daw, Gershman, Seymour, Dayan, & Dolan, 2011). This model-agnostic signature comprises the proportion of engaging the switch or stay behavior that is predicted by model-based control following rare state transitions. Model-based control predicts one should switch more often from one's prior action if a state to which a rate transition led was rewarded, because one is more likely to reach the rewarded state via the action one did not take last trial. For the same reason, model-based control predicts one should stay with one's prior action more often if a rare-transitioned state was not rewarded. Model-basedness is thus computed via the following equation, where ‘#’ denotes a tally:6
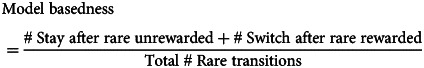


### Relating compulsivity to state transition learning

We characterized state transition learning in each of the 1413 participants from Gillan et al. (2016) by estimating the state transition learning rate (*γ*) in the ISTL model that best fitted participants' choices. We then regressed compulsivity scores on the state transition learning rate as well as covariates of age, sex, and IQ in line with Gillan et al. (2016). We then tested whether the use of transition knowledge for planning (*β*_MB_) mediated the aforementioned relation between *γ* and compulsivity. Due to a nonlinear relation between *β*_MB_ and *γ*, we log-transformed each to more fully account for shared variance (online Supplementary Fig. S5).

### Study 3: quantifying disruptions in state transition learning when transitions change

To determine whether the finding of a compulsivity-related impairment in state transition learning was specific to environments with stable state transitions, we analyzed behavioral data from an additional learning task wherein state transitions changed throughout the task. This task was a component of a larger study (*n* = 192; Sharp et al., 2021) designed primarily to measure how individuals switch between punishment avoidance and reward pursuit goals when learning from reinforcement. Importantly, we assayed participants in this study for compulsivity using the same measure as utilized from Study 1, the OCI-R (Foa et al., 2002). Here, we describe relevant details required to understand how the task probed state transition learning. Full details of Study 3's task and hypotheses can be found in Sharp et al. (2021; Fig. 1).

To win points, participants had to learn from experience the probabilities that each of two actions (pressing ‘*j*’ or ‘*g*’) would transition them to each of two ‘states’ (presented as colored circles). Importantly, the four transition probabilities slowly drifted across the task according to independent random walks. After choosing an action, participants could reach any combination of states – none, one, or both. Thus, it was possible to reach two states simultaneously, or reach neither state. Participants were presented with an instructed goal each trial: either to seek the ‘reward’ state (i.e. the gold circle) as getting to this state would reward the participant with 1 point, or to avoid the ‘punishment’ state (i.e. black circle) as reaching this state would cost the participant 1 point. Reaching a state not referred to in the instructions had no consequence in terms of winning or losing points. However, observing whether the chosen action led to a presently irrelevant state could, via state transition learning, guide choices in subsequent trials wherein instructions made this state relevant. This aspect of the task allows an evaluation of state transition learning that is separate from value learning.

Given that state transitions could not be inferred from explicit instruction in this task, but rather had to be learned via experience, in line with prior work (Dayan, 1993; Russek et al., 2017) we modeled state transition learning as an associative, incremental learning process. Note, this learning process is identical to how state transition learning was modeled in Study 2:7
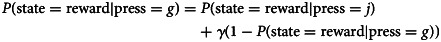


In this example, the model updates its estimate of the probability that pressing ‘*j*’ leads to the ‘reward’ state after having just experienced that state transition. All state transitions probabilities are updated in this way every time a state transition is observed.

To choose an action, an agent computes the expected value of each action by multiplying the state transition probability with the values of the possible outcome states, as defined by the trial-specific, instructed goal. Here, the agent is facing an avoid punishment trial, for which reaching a black punishment state results in a loss of 1 point (i.e. a value of −1) and reaching a reward gold state delivers no points and is thus irrelevant. Actions values for pressing ‘*g*’ are thus:
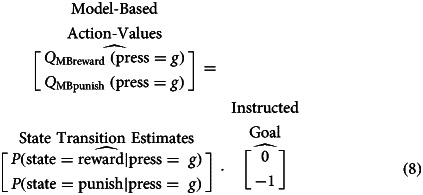


Note the model-based action-value for reward 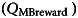 equals 0 above, as determined by the 0 in the ‘Instructed Goal’ vector.

In the best-fitting model for Study 3, three strategies influence decision-making in addition to a model-based strategy. A goal-perseveration strategy uses model-based state transition estimates to always both avoid a punishment state (*Q*_GPpunish_) and seek the reward state (*Q*_GPreward_). Thus, goal-perseveration values are computed by setting the goal vector in Eq. 9 to 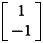. A model-free strategy computes action-values (*Q*_MF_ ) solely based on points gained or lost previously in response to each action, thus neglecting state transitions and the presently instructed goal. Finally, an action perseveration strategy consists of a bias (*Q*_AP_) to stay with the same action taken at the last trial irrespective of any task feature.

The action-values computed by each strategy are integrated via a weighted sum. Thus, each action value is multiplied by weights (e.g. *β*_MBpunish_ and *β*_MBreward_) that reflect the degree to which the model utilizes the corresponding strategy. For the MB and GP strategies, these utilization weights can differ in strength for reward seeking and punishment avoidance. For the action ‘press = *g*’ (which we omit from the right side of the equation for concision) the weighted sum is computed as:9
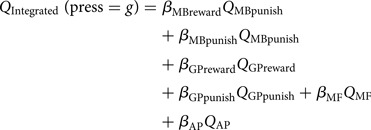


The integrated action value (*Q*_Integrated_ (press = *g*)) is then inputted into a softmax function to generate the policy:10
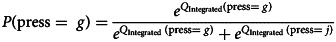
11
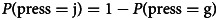


Since model-based utilization in this task differed across reward pursuit and punishment avoidance goals, we included both *β*_MBreward_ and *β*_MBpunish_ in subsequent regression analyses to determine how each were related to compulsivity. Importantly, an extensive model comparison showed that the model delineated above outcompeted alternative models that used only a subset of these decision strategies as well as several variations within them (Sharp et al., 2021 for full details).

## Results

In the one-step revaluation task, a subject was deemed to have successfully learned state transitions for a given block of learning if they chose both actions that had the highest probability of transitioning them to the two states presented at test. Examination of the data showed participants learned the correct state transitions in the majority of cases. On average, participants performed correctly on 3.22 out of 5 conditions (*N* = 174, s.e.m. = 0.11), significantly greater than chance level (chance = 1.25, *t*_173_ = 17.9, *p* < 0.001).

### Compulsivity is associated with poor state transition learning

**Fig. 2.**
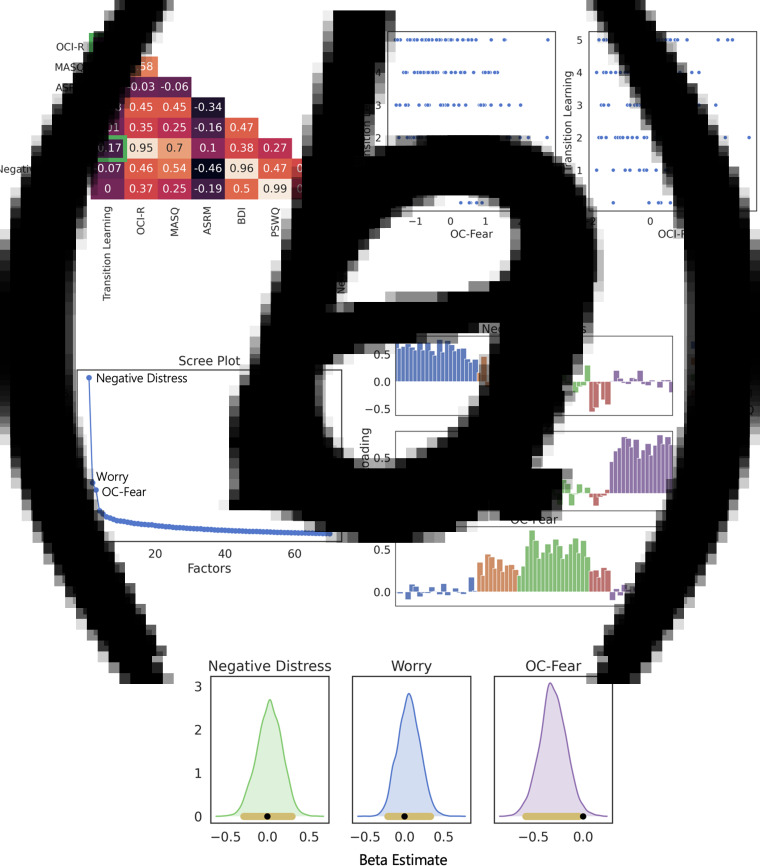
Study 1: transition learning and psychopathology. (a) Correlations between psychopathology dimensions, transdiagnostic factors, and choice behavior. The left plot comprises correlations between psychopathology dimensions (denoted by their associated questionnaire's abbreviation), transdiagnostic factors derived from exploratory factor analysis, and how well participants learned state transitions. We outline in green the two significant associations involving transition learning in the task, and plot the raw data comprising these correlations in the middle and right plots (*p* < 0.05). (b) Exploratory factor analysis of psychopathology dimensions. Left is the scree plot showing that variance explained plateaus after the first three factors. The eigenvalues for the three components were 16.70, 5.50, and 4.70 for the Negative Distress, Worry, and OC-Fear factors, respectively. The bottom bar plots show the composition of each of these factors in terms of the factor loading of each individual question from all five questionnaires used. Each questionnaire is denoted by a different color. Note, the dataset met both the Bartlett's test of sphericity (*p* ≤ 0.001) and Kaiser-Meyer-Olkin test of sampling adequacy (KMO = 0.83). (c) Posterior density plots estimating the effects of the three latent psychopathology factors on transition learning. The black bar denotes the ROPE and the yellow bar the 95% HDI. Only the OC-Fear components has the ROPE entirely outside its HDI. The width of the ROPE was defined as 10% of the standard deviation of the posterior distribution (i.e.±0.01).

Rank correlations between psychopathology dimensions and state transition learning showed that OCD symptoms, as measured by the OCI-R, were significantly related to worse transition learning (*r* = −0.154, *p* = 0.04; Fig. 2a). Given that this analysis could not account for co-morbidities between symptoms, we next sought to employ a transdiagnostic analysis that could reveal the contribution of additional symptoms to weakened state transition learning.

To account for psychopathological co-morbidities (Sharp, Miller, & Heller, 2015), and thus potentially improve our ability to capture underlying neurocomputational dysfunctions (Norbom et al., 2019), we conducted an exploratory factor analysis (Fig. 2b) on the set of questionnaire items we administered. A three-factor structure was implied by visual inspection of the scree plot. Parallel analysis supported a strategy of *not* reducing this number of factors, which aligns with prior work using similar questionnaires (Gillan et al., 2016). The three components of interest can largely be interpreted as (1) negative distress, (2) worry, and (3) obsessive-compulsive and fear (OC-Fear; see Fig. 2b). This result agrees with recent work that also found a factor comprising OC and fear/panic symptoms (Wise & Dolan, 2020), as well as with a wealth of psychometric literature that used exploratory factor analytic approaches and demonstrated an overlap between OCD and fear/panic symptomatology (Stasik, 2014; Stasik, Naragon-Gainey, Chmielewski, & Watson, 2012).

Out of the three transdiagnostic factors, only the OC-Fear factor significantly correlated with worse state transition learning (*r* = −0.17, *p* = 0.03; Fig. 2a). We additionally examined how all three factors relate to state transition learning within a single hierarchical Bayesian logistic regression model. The results again showed that only the OC-Fear factor (95% HDI −0.58 to −0.05, mode = −0.33) significantly related to poor state transition learning (Fig. 2c).

### The impact of transition learning on model-based decision making

**Fig. 3.**
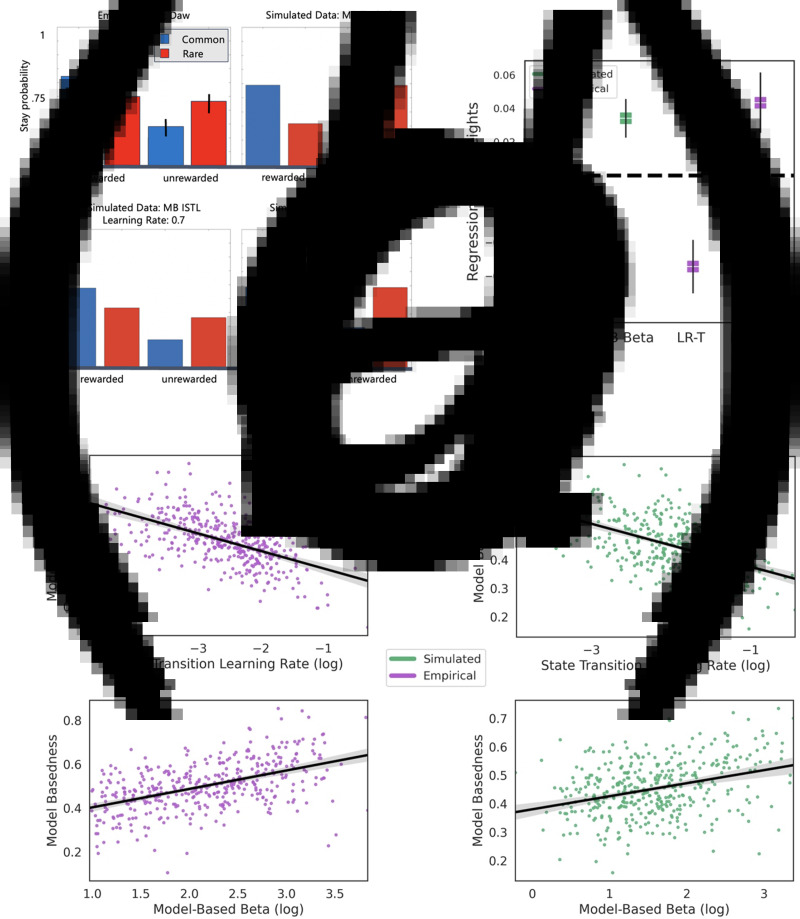
Study 2: state transition learning rate and model-based behavior in the two-step task. (a) A high state transition learning rate can produce behavior resembling compromised model-based control. The plots show the proportion of times a participant (real or simulated) engaged the same action they deployed in the preceding trial (‘Stay probability’) conditioned on whether or not one's prior action was followed by a common or rare state transition and whether reward was administered. The behavioral signature of model-basedness is shown in the top right plot (Simulated Data: MB Typical). The deviation of Daw et al.'s real data (top left panel) from this signature was deemed to reflect reduced utilization of intact transition matrix knowledge when making decisions, relative to a competing model-free system. The bottom row depicts simulations of the ISTL algorithm which gradually learns the transition matrix from experienced state prediction errors. The bottom left plot shows that the same qualitative pattern found in Daw et al.'s empirical data (top left) can emerge due to a fast transition learning rate, even in the absence of a putative model-free system. (b) Model-basedness as a function of transition learning rate and model-based *β* in empirical data. Model-basedness quantifies the degree to which participants complied with the behavioral signature of model-based choice as shown in panel A (e.g. switching after a rare transition was rewarded; see Methods). The top subplot reflects how model-basedness inversely covaries with state transition learning rate whereas the bottom subplot shows the positive relationship between model-basedness and model-based *β*. Both were generated using a subsample of participants with high (>2.5) model-based control. (c) Model-basedness as a function of transition learning rate and model-based *β* in simulated data. As a post-predictive check, we simulated the data using the winning ISTL model and best-fitting parameters, which generated the same effects as the empirical data in (c). (d) Regression weights of computational parameters in explaining model-basedness.

To test more specifically how state transition learning is disrupted in the context of model-based control, we modeled choice data (*n* = 1413; Gillan et al., 2016) from the two-step task using the ISTL model. We first used this model to determine if past signatures of reduced model-based control could be accounted for by a disruption in incremental state-transition learning. For this purpose, we simulated data by having the model ‘play’ the task with different transition learning rates, and fixing other parameters to fitted group means. In so doing, we show that having a suboptimal high state transition learning rate reproduces the empirical effect reported in Daw et al. (2011), which was deemed to reflect decreased model-based planning (Fig. 3a).

To determine whether participants learned state transitions incrementally or via counting, and whether they differed from one another in their transition learning rates, we conducted an extensive comparison between different computational models in terms of how well each model explained participants' choices (online Supplementary Fig. S3). We found that participants' choices were best explained by the ISTL model, which features an individually fit incremental learning rate for state transitions (log Bayes factor = 274.78 compared to the Typical model used to analyze two-step task data). Model recovery tests demonstrated our analysis successfully recovered the true model from simulated data, validating our model selection procedure (online Supplementary Note 1).

To determine whether differences among participants in model-based control reflected differences in state transition learning rate (*γ*) and in the use of transition knowledge for making choices (*β*_MB_, ‘model-based beta’), we fitted the values of these two parameters of the computational model to each participants' choices, and we regressed onto them a model-agnostic behavioral signature of model-based control. This signature (‘model-basedness’) tallies optimal switch behavior that should occur after participants experience rare transitions. We found that the model-based *β*, *β*_MB_ (*β* = 0.01, s.e. = 0.003, *p* = 001) and the state transition learning rate, *γ* (*β* = −0.09, s.e. = 0.006, *p* < 0.001) each predicted unique variance in model-based behavior.

Given that the recoverability of *γ* was expectedly low when *β*_MB_ was itself very small, we computed the same regression on a subsample of participants (*n* = 409) with a relatively high *β*_MB_ (*β*_MB_ > 2.5). We determined this threshold for *β*_MB_ by using increments of 0.5 and selecting the threshold that maximized parameter recoverability of state transition learning rate while also ensuring there was no significant difference in the mean level of compulsivity across the reduced and full samples (mean difference = −0.06, s.d. = 0.93, *t*_408_ = 1.80, *p* = 0.07). Enhanced recoverability when excluding low-*β*_MB_ participants manifested in a substantial increase in the correlation between estimated and true *γ* in simulated data (*r* = 0.61 compared to *r* = 0.22; difference between correlations: *Z*_408_ = 6.46, *p* ≤ 0.001). The regression results for this subset of participants agreed with the results for the full sample (*β*_MB_: *β* = 0.043, s.e. = 0.009, *p* < 0.001 and *γ*: *β* = −0.054, s.e. = 0.008, *p* < 0.001; Fig. 3b). Finally, we further confirmed the ability of the model to explain model-basedness in the empirical data using a posterior predictive check wherein we replicated the empirical effects noted above in synthetic data (*n* = 400) generated from the ISTL model and individual-level best-fitting parameters (*β*_MB_: *β* = 0.34, s.e. = 0.006, *p* < 0.001 and *γ*: *β* = −0.068, s.e. = 0.007, *p* ≤ 0.001; Fig. 3c).

### In a stable environment, high-compulsivity participants update state-transition estimates too fast

To determine whether compulsivity is related to state transition learning (*γ*), we regressed the former onto the latter, controlling for age, gender, and IQ. This showed that state transition learning and compulsivity were positively related (*β* = 0.15, s.e. = 0.05, *p* = 0.003). Next, replicating the core finding in Gillan et al. (2016), we showed that utilization of transition knowledge (*β*_MB_) was inversely related to compulsivity (*β* = −0.12, s.e. = 0.03, *p* < 0.001). Whereas *β*_MB_ decreased with compulsivity, *γ* increased with compulsivity. Thus, that both were related to compulsivity cannot be explained by an identifiability tradeoff between them, since this (anticorrelated) tradeoff would have made both parameters share the same relationship with compulsivity.

**Fig. 4.**
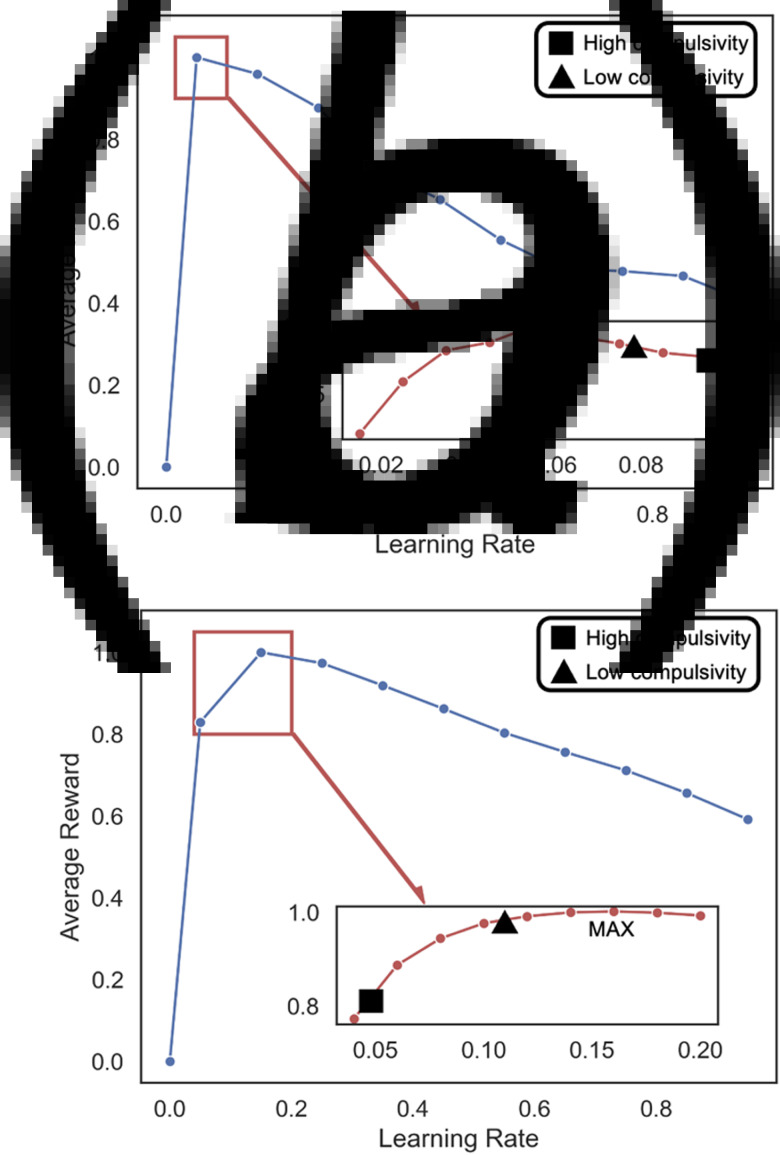
Compulsivity-associated state transition learning rates are suboptimal. (a) Compulsivity is associated with sub-optimally fast state transition learning in a stable environment (Study 2). We simulated agents that played the exact same task as described in Gillan et al. (2016) and plot the min-max normalized average reward. We instantiated agents with a model-based *β* weight that maximized reward earned but was still within the tail of the empirical range, using a selection procedure for extreme values in skewed distributions (Rousseeuw & Hubert, 2011). We set all other parameters to their group-fitted medians for distributions that were highly skewed (*z*-score >4, which was the lowest among statistically significant *z*-scores), and group-fitted means otherwise. Agents played the game 100 000 times with different state transition learning rates [sampling from (0,1) in increments of 0.1; main plot], and 1 000 000 times within a region of interest around the optimal and empirical learning rates (denoted by red box) for increased precision. The low- and high-compulsivity groups included participants scoring <–1 and >1 on the standardized scale of compulsivity factor derived in Gillan et al. (2016). Medians from each group (due to their skew) are plotted on the inset plots. (b) Compulsivity is associated with sub-optimally slow state transition learning in a changing environment (Study 3). Plots were generated using the procedure described in panel A, here applied to the Study 3 model.

In a given task, a state transition learning rate that is too high or too low may result in misestimations of the true state transition probabilities. Thus, we next asked whether the higher state transition learning rates associated with compulsivity were suboptimal or well-suited to the experimental task. To test this, we simulated agents with varying state transition learning rates, while setting *β*_MB_ to a high value (within the empirical range) and all other parameters to group-fitted means. Each agent played the task 1 000 000 times and the average reward for each state transition learning rate was computed. The results showed that the higher learning rates associated with compulsivity were suboptimal, in the sense that they made the simulated agent win less reward (Fig. 4a).

Subsequently, we sought to determine whether the two parameters, state transition learning rate (*γ*) and model-based beta (*β*_MB_), independently contribute to explaining compulsivity, or whether, as proposed by Seow et al. (2021), variations in *γ* can account for known compulsivity-related deficits in *β*_MB_. Consistent with the latter possibility, we found that *γ* anticorrelated with *β*_MB_ (*β* = −0.98, s.e. = 0.04, *p* < 0.001). Thus, as all three variables were correlated with one another, we tested for mediation by regressing compulsivity on both *β*_MB_ and *γ* in the same model. This demonstrated that *β*_MB_ (*β* = −0.10, s.e. = 0.03, *p* = 0.001) fully mediated the relationship between *γ* and compulsivity (*β* = 0.05, s.e. = 0.06, *p* = 0.40). To further validate this result, we additionally performed the mediation analysis on a subsample of participants (*n* = 409) that included only participants with a *β*_MB_ estimate of 2.5 or greater, for which the true learning rate is more accurately recovered. We again found that *γ* was associated with compulsivity (*β* = 0.14, s.e. = 0.06, *p* = 0.02) and this relationship was fully mediated by *β*_MB_ (*β*_MB_: *β* = −0.23, s.e. = 0.09, *p* = 0.006; *γ*: *β* = 0.0004, s.e. = 0.08, *p* = 0.99).

### In a changing environment, high-compulsivity participants update state-transition estimates too slowly

To determine whether a compulsivity-related disruption in state transition learning is due to learning too quickly or due to a failure in adapting the rate of state transition learning to the pace at which state transitions change, we next examined learning about continuously changing state transitions. To do so, we re-analyzed data from a recently completed study in our lab (Sharp et al., 2021) in which participants were required to learn changing state transitions in order to pursue trial-specific goals [see Methods, Study 3 for brief task and model descriptions; Sharp et al. (2021) for in-depth details of task and model comparison]. Unlike in Study 2, here we found that compulsivity was associated with lower state-transition learning rates (i.e. *γ*; *β* = −1.69, s.e. = 0.8, *p* = 0.035).

To determine whether such slow state-transition learning was detrimental to task performance, as in Study 2, we simulated task performance and computed the average reward that could be earned with various state transition learning rates. This showed that the lower state transition learning rates associated with compulsivity were suboptimal (Fig. 4b). Thus, in a changing environment, which warrants faster learning than a stable environment, individuals with compulsivity update their estimates of state transitions at too slow a rate.

In this study, neither *β*_MB_ parameter was significantly associated with compulsivity. However, it is important to stress that Study 3's task involved explicit instruction on a trial-by-trial basis to prompt model-based flexibility, which has been shown to significantly increase model-based control, and reduce between-subject variance in this regard (da Silva & Hare, 2020). Thus, *β*_MB_ values found here may not be comparable with those found in prior studies.

## Discussion

We showed in Study 1 that compulsivity was associated with impaired learning of state transitions in a novel revaluation task that was designed to isolate the effects of transition learning. Additionally, Study 1 found preliminary evidence that state transition learning deficits are associated with a transdiagnostic factor which is influenced in part by symptoms of fear. We next demonstrated across two independent studies that compulsivity is related to state transition learning impairments, in both one- and two-step decision tasks, and in the presence of stable or changing state transitions. In Study 2, we showed that when state transitions are stable, compulsivity is associated with a sub-optimally high state transition learning rate, whereas Study 3 demonstrated that when state transitions change, compulsivity is associated with a sub-optimally low state transition learning rate.

These findings extend recent evidence that compulsivity is associated with greater uncertainty regarding state transitions (Fradkin, Adams, Parr, Roiser, & Huppert, 2020a, 2020b; Seow et al., 2021). For example, previous work implicated disruptions in uncertainty-related learning of shifting cue-outcome contingency (Fradkin, Ludwig, Eldar, & Huppert, 2020b). Here, our finding that compulsivity-related disruptions in the rate of state-transition learning depended on whether state transitions changed over the course of the task suggests that individuals with compulsivity have trouble estimating higher-order statistics, such as state transition volatility (e.g. Behrens, Woolrich, Walton, & Rushworth, 2007). Future work could seek to manipulate the rate of change of state transitions, within task, in order to determine if the aforementioned hypothesis can parsimoniously explain the present results as well as related findings in prior work (e.g. Fradkin et al., 2020a).

One caveat to Study 1's results is that they do not account for multiple comparisons across the different measures provided by the one-step revaluation task. However, the possibility of a false-positive is mitigated by the results of Study 2 and Study 3, which offer two ‘conceptual replications’ of Study 1's core finding – that compulsivity is associated with disrupted state transition learning. A conceptual replication tests the same hypothesis using different task designs to determine whether the core hypothesis is robust under task variation (Watts, Duncan, & Quan, 2018). It has been argued that a conceptual replication is one of the most effective ways to rule out false-positive findings (Crüwell et al., 2019).

By contrast, findings related to fear symptoms as captured by an anxious arousal measure were only implicated by Study 1 and thus should be considered preliminary. Nevertheless, we note that our finding of an OC-Fear factor aligns with recent psychometric literature and online computational psychiatry studies in large samples (Levin-Aspenson, Watson, Ellickson-Larew, Stanton, & Stasik-O'Brien, 2021; Stasik, 2014; Wise & Dolan, 2020) showing, across several factor analytic approaches, that fear and OCD symptoms converge. This is unsurprising given that, in many instances, fear of an aversive outcome (e.g. contamination) may partly be the cause of compulsions. That said, future work should determine if fear-related impairments in transition learning are entirely driven by compulsivity, especially given that compulsivity scores loaded more strongly onto our OC-Fear factor. In any case, this finding does not contradict a previously observed lack of association between anxiety and model-based control (Gillan et al., 2020b; Heller, Ezie, Otto, & Timpano, 2018), given the former studies did not specifically assay fear symptoms as distinct from classical anxiety symptoms (e.g. worry). Indeed, worry in Study 1 was not associated with state transition learning deficits.

The factor analysis employed in Study 1 was limited by the sample size afforded by study resources, which entailed a low item:participant ratio. Additionally, we could not mitigate this problem by performing the factor analysis on subscales (Gillan et al., 2020a, 2020b), as only one of five questionnaires was comprised of subscales. Thus, validation of the specific factor structure found and its relation to state transition learning would require direct replication on a larger sample size.

Finally, it is important to ask why the mediational hypothesis (Seow et al., 2021), that reduced utilization of model-based control mediates a relation between poor transition learning and compulsivity, was only found in Study 2. First, Study 3 included a one-step task wherein utilization of model-based control is potentially less effortful to leverage than in the two-step task. Second, and more importantly, Study 3's task included trial-by-trial instruction that repeatedly signaled to participants the need for model-based control. Prior work has shown that such instruction greatly improves model-based control (da Silva & Hare, 2020).

Future work should determine how other aspects of the utilization and consolidation of cognitive maps, such as episodic retrieval (Talmi, Lohnas, & Daw, 2019) and offline replay of trajectories (Eldar, Lièvre, Dayan, & Dolan, 2020), are affected in compulsivity. Finally, developmental work is crucial to understand whether a state transition learning deficit emerges before compulsive symptomatology, or whether it is an effect of this pathology.

## Supplementary material

For supplementary material accompanying this paper visit https://doi.org/10.1017/S0033291721003846. click here to view supplementary material
