# Supplementary material for: Disrupted state transition learning as a computational marker of compulsivity
Source: Psychol Med. 2021 Sep 24;53(5):2095–105. doi: 10.1017/S0033291721003846 (PMC10106291; doi:10.1017/S0033291721003846)
Supplement: Supplementary file 1 [file S0033291721003846sup001.pdf]

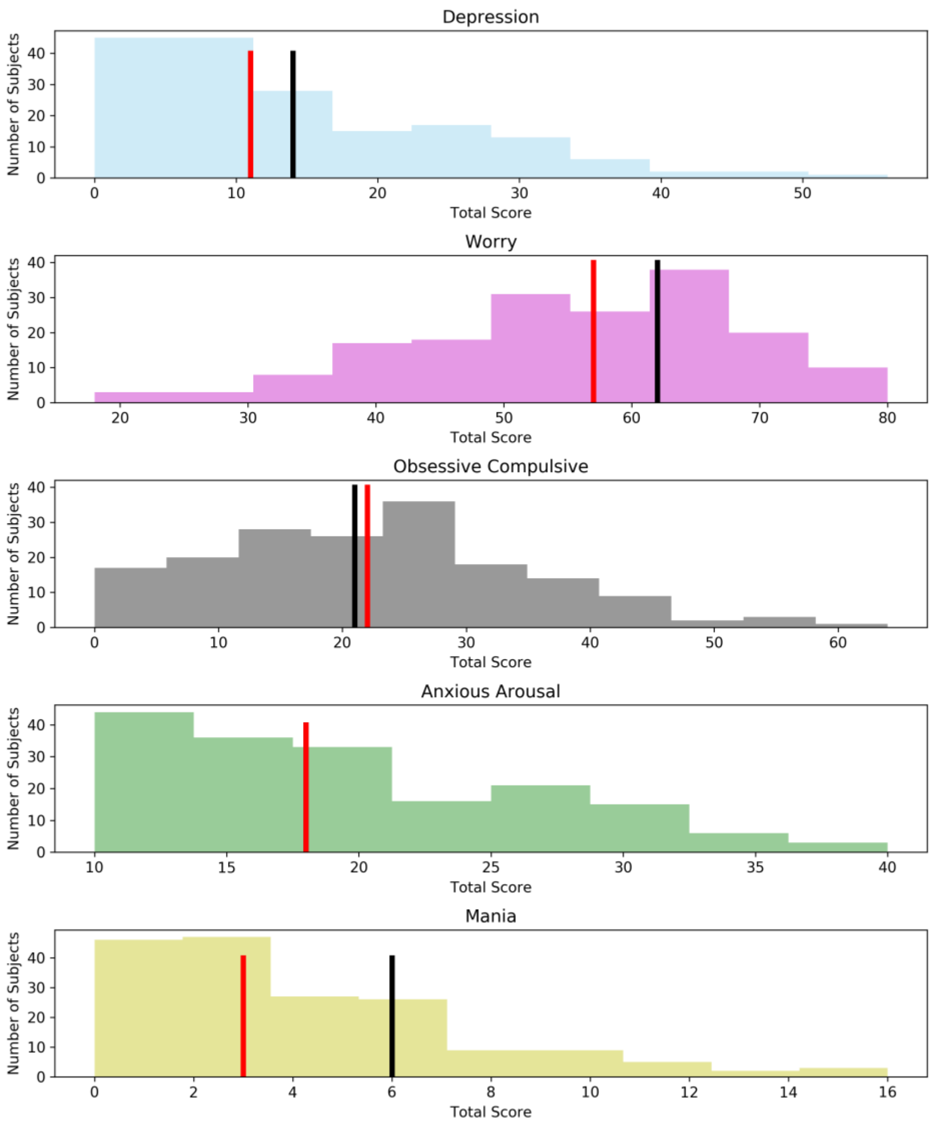

Figure S1. Self-report distributions. Frequency plots for each self-report measure used to assay various pathologies. In each plot, the black bar denotes cut-offs of mild but clinically-significant symptoms, and the red bar denotes the median. Note that for most measures, the median is either slightly below or above the clinical cut-off; only mania has a large gap between the two. Cut-offs for pathological worry (cutoff=62) was determined in Curtiss and Klemanski (2015), for depression (cutoff-14 for at least mild depression) in Beck et al. (1996), for obsessive-compulsive symptoms (cutoff=21) in Foa et al. (2002) and mania (cutoff=6) in Altman et al. (1997). We used the median to define the MASQ-AA short form clinical cutoff (cutoff=18) which is close to a very similar questionnaire, the MASQ-D30 Anxious Arousal subscale (cutoff=17).

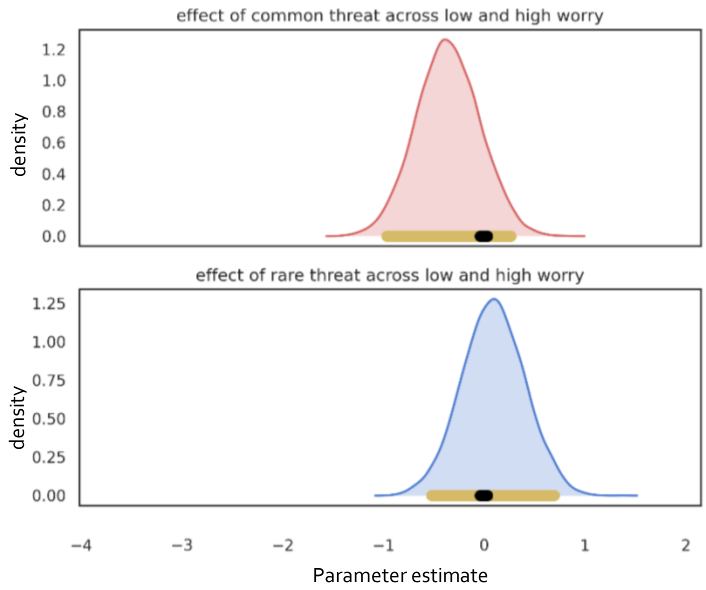

Figure S2. Interaction between condition effect and psychopathology. In each plot, the terms "positive" or "threat" denote whether or not a positive or negative image was interposed between state transitions, and the terms "common" or "rare" denote the probability of the state transition (common = 50%, rare=30%; the remaining state transition was of non-interest because both actions led to the final state 20% of the time) No interaction approached a significant effect, as noted by the ROPE being near the mode of each interaction effect. We additionally tested whether differences between condition (e.g., common positive – rare positive) depended on psychopathology group, all of which were non-significant.

S3. Logic of model step-wise model testing and full model descriptions

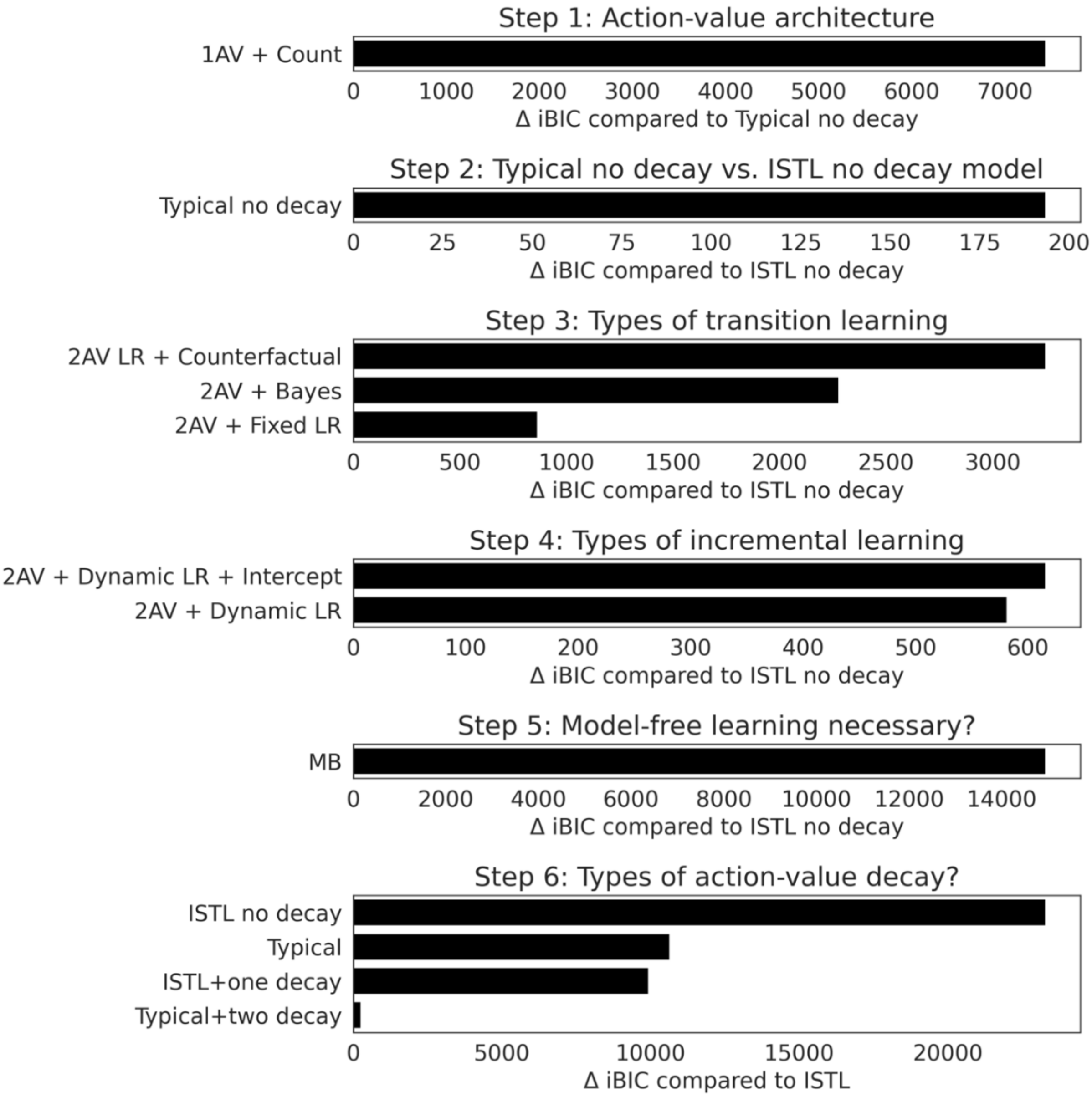

Figure S3. We conducted a step-wise model comparison, where each step is denoted by the box next to each subplot. Each subplot shows iBIC difference of each tested model compared to the best-fitting model (the one with the lowest iBIC.

We fit the models to actual data from Gillan et al. (2016) in a step-wise fashion. We will first describe the logic behind each step. We then list in full detail the model formalisms.

We first compared two standard learning architectures from the literature, wherein differences between the two models are reflected in how first-stage actions are learnt. One model combines two prediction errors concerning first-stage actions (after reaching stage 2 and after receiving a reward) into a single action value (1AV model) where the second prediction error is weighted by an eligibility trace. The second encodes two separate first-stage action values (which we call the "typical no decay" model, as it was used in Gillan et al., 2016), one value for each type of prediction error (Step 1), and two separate parameters control the influence of these values over first-stage actions. At the conclusion of this step, it was apparent that keeping two separate first-stage action values (i.e., typical model) provided the best fit to the data.

We thus tested whether the 'ISTL no decay' learning architecture would be enhanced by the inclusion of incremental state transition learning with individual learning rates (Step 2). For this purpose, we compared the 'typical no decay' model, which learns transitions via a counting rule, to a model that learns transitions from state prediction errors multiplied by a subject-specific transition learning rate, Incremental State Transition Learning model (ISTL). The results showed that subjects' behavior was best captured by a model with between-subject variation in the rate of incremental transition learning from experienced transitions.

In step 3, we included three alternative ways in which state transition learning might differ from the incremental state transition learning model: a model where the learning rate does not vary across subjects ('2AV +Fixed LR'), a model where transition learning is realised via Bayesian inference ('2AV + Bayes'), and a model where transitions for actions not taken are updated via counterfactual inference ('2AV + Counterfactual'). These alternative models did not explain the data as well as a model that allowed variation between subjects in learning rate (ISTL no decay).

We then determined whether transition learning rates decrease as information is accumulated, as expected from an optimal observer (Step 4). Thus, we compared the so far best-fitting model with models where learning rates decay over time to 0 ('2AV + Dynamic LR') or to some fixed learning rate ('2AV + Dynamic LR + Intercept'). These dynamic LR models did not explain the data as well as a model that allowed variation between subjects in learning rate, but within subjects kept learning rates constant (ISTL no decay).

Next, since the behavioural signatures characterizing average performance could be generated by a pure model-based learner (Step 5), we tested whether model-free learning was at all necessary to explain the choice data. For this purpose, we compared ISTL to an 'MB' learner that only used model-based inference (and perseveration) to determine first-stage actions. Again this did not explain the data as well as the ISTL no decay model.

Finally, we tested a set of models in which learned information decays over trials. As reported in Gillan et al. (2016), decay may allow the model to better explain participants' behavior. Thus, we first compared the ISTL model without decay to the original Gillan et al. (2016) model, which we refer to as the 'Typical' model. In this model, values of actions not chosen decay with a rate of 1-learning rate. Secondly, we enhanced the Typical model by including the same incremental state transition learning process that is included in the ISTL no decay model, allowing also state transitions to decay at rate of 1-state transition learning rate ('ISTL+one decay). Last, we tested

variant of these two models wherein values of unchosen actions decayed at a rate that constituted a separate free parameter (‘Typical + two decay’ and ‘ISTL’, respectively). The ISTL model explained the data the best of all models.

Model descriptions

July 16, 2021

1 Models with decay on unchosen actions

1.1 Gillan et al 2016 model = Typical model

The following model includes two updates in the model-free system for first-stage actions: namely a Temporal Difference update (known as TD(0)) and a Monte Carlo update. The Monte Carlo update reinforces first-stage action values according to the final reward only. Note that the Monte Carlo update is equivalent to lambda=1 in Daw (2011).

Allowing each of these possible model-free updates to influence first-stage action learning was carried out in Gillan et al. (2016). The first action-value represents the prediction of which second-stage one will arrive in, each of which has its own value depending on how rewarded it has been in the recent past. The second first-stage action value represents the prediction that the first-stage action will be rewarded after the second-stage choice. Separating these first-stage action values in turn removes the requirement for an eligibility trace. All models use the Bellman equation to derive model-based action values.

1.1.1 Variables

Below, t=time, s=state, a=action. At stage one, two images appeared, one of which could be selected with a given action. The image was always chosen with a certain action that did not change across the task. Each action led to two possible states (determined by a transition matrix), wherein each state had two unique images. At this second stage, an image again is selected with a given action. Note only an "s" subscript is used for second-stage action values, since one could either transition to state 2 or 3. The selection of this image would lead to a monetary reward determined by a latent probability that drifted across the task (see Gillan et al., 2016).

1.1.2 NOTE

See Gillan et al. (2016) for how the inverse temperature parameters are rescaled by learning rate which improves parameter estimation. Actions taken are denoted as 'a' and unchosen actions as 'u'. Second stage states arrived at are denoted as 's' whereas states not arrived at are denoted as 'x'.

R = reward

$$T = \begin{bmatrix} P(s=1|a=1) & P(s=1|a=2) \\ P(s=2|a=1) & P(s=2|a=2) \end{bmatrix} \text{ Transition matrix}$$

M = one-hot vector indicating which first-stage action was taken on the last trial.

First-stage action values: $Q_{MF0_{t,a}}$ = First-stage action value predicting value at second stage.

$Q_{MF1_{t,a}}$ = First-stage action value predicting reward after second stage.

$Q_{MB_{t,a}}$ = Model-based value of action 1

Second-stage action values: $Q_{MF2_{t,s,a}}$ = Second-stage action value predicting reward.

1.1.3 Free Parameters

All alpha parameters below were drawn from Beta priors that spans the 0-1 range.

$1 - \alpha$ = decay rates on chosen and unchosen actions

All beta parameters below were drawn from Gamma priors that spans all positive real numbers.

β_{MF0} = inverse temperature for Q_{MF0} at first stage.

β_{MF1} = inverse temperature for Q_{MF1} at first stage.

β_{MB} = inverse temperature for Q_{MB} at first stage.

β_{st} = strength of perseveration at first stage. This multiplies the M vector, which the previously enacted first-stage action.

β_{MF2} = inverse temperature for Q_{MF2} at second stage.

1.1.4 Learning computations

- Updating the transition matrix:

Each trial, a transition counter is updated. For example if state1, action1 led to state 2 once, and on the next transition, the same transition occurs, the counting matrix would be updated as follows:

$T_{counting} = \begin{bmatrix} 1+1 & 0 \\ 0 & 0 \end{bmatrix}$

T can be one of two matrices at and given trial $T_1 = \begin{bmatrix} 0.7 & 0.3 \\ 0.3 & 0.7 \end{bmatrix}$ or $T_2 = \begin{bmatrix} 0.3 & 0.7 \\ 0.7 & 0.3 \end{bmatrix}$ or $T_3 = \begin{bmatrix} 0.5 & 0.5 \\ 0.5 & 0.5 \end{bmatrix}$ at any given trial.

This is determined by the $T_{counting}$ matrix. When $T_{counting}(1,1) + T_{counting}(2,2) > T_{counting}(1,2) + T_{counting}(2,1)$, then T_1 is used. When the inequality is the converse, then T_2 is used. If they are equal, then T_3 is used.

- Updating chosen action-values

$Q_{MF0_{t+1,a}} = Q_{MF0_{t,a}}(1 - \alpha) + Q_{MF2_t}$

$Q_{MF1_{t+1,a}} = Q_{MF1_{t,a}}(1 - \alpha) + R$

$Q_{MF2_{t+1,s,a}} = Q_{MF2_{t,s,a}}(1 - \alpha) + R$

- Decaying unchosen actions

$Q_{MF0_{t+1,u}} = Q_{MF0_{t,u}}(1-\alpha)$

$Q_{MF1_{t+1,u}} = Q_{MF1_{t,u}}(1-\alpha)$

$Q_{MF2_{t+1,s,u}} = Q_{MF2_{t,s,u}}(1-\alpha)$

$Q_{MF2_{t+1,x,a}} = Q_{MF2_{t,x,a}}(1-\alpha)$

$Q_{MF2_{t+1,x,u}} = Q_{MF2_{t,x,u}}(1-\alpha)$

Model-based q-values are then computed via the Bellman equation:

$Q_{MB_{t+1}} = P(s_1|a_i) \max_{a\in\{1,2\}} Q_{MF2}(s_1, a_i) + P(s_2|a_i) \max_{a\in\{1,2\}} Q_{MF2}(s_2, a_i).$

1.1.5 Decision computations

First-stage action:

$P(a) \propto e^{(\beta_{MF0}Q_{MF0}+\beta_{MF1}Q_{MF1}+\beta_{MB}Q_{MB}+\beta_{st}M)}$

Secon-stage action:

$P(a, s_i) \propto e^{(\beta_{MF2}Q_{MF2})}$

1.2 Typical + two decay

Same as typical model except an extra free parameter, ϵ, decays actions that were NOT chosen

- Updating chosen action-values

$Q_{MF0_{t+1,a}} = Q_{MF0_{t,a}}(1-\alpha) + Q_{MF2_t}$

$Q_{MF1_{t+1,a}} = Q_{MF1_{t,a}}(1-\alpha) + R$

$Q_{MF2_{t+1,s,a}} = Q_{MF2_{t,s,a}}(1-\alpha) + R$

- Decaying unchosen actions at a rate defined by “D” bounded between [0,1]

$Q_{MF0_{t+1,u}} = Q_{MF0_{t,u}}(D)$

$Q_{MF1_{t+1,u}} = Q_{MF1_{t,u}}(D)$

$Q_{MF2_{t+1,s,u}} = Q_{MF2_{t,s,u}}(D)$

$Q_{MF2_{t+1,x,a}} = Q_{MF2_{t,x,a}}(D)$

$Q_{MF2_{t+1,x,u}} = Q_{MF2_{t,x,u}}(D)$

1.3 ISTL + one decay

Here we simply amend the Typical model used in Gillan et al. (2016) except instantiate state transition learning in an incremental process.

γ = learning rate for state transitions

- Updating the transition matrix:

$T = \begin{bmatrix} P(s=1|a=1) & P(s=1|a=2) \\ P(s=2|a=1) & P(s=2|a=2) \end{bmatrix}$

Each trial, a transition estimate is updated with a learning rate, and probabilities are at that time normalized. For instance, if action 1 is taken and transition to state 2:

$P(s=2|a=1)_{t+1} = P(s=2|a=1)_t + \gamma(1 - P(s=2|a=1)_t)$

and

$PP(s=1|a=1)_{t+1} = 1 - P(s=2|a=1)_{t+1}$

1.4 ISTL model

The final and winning ISTL model included the same incremental state transition learning model in ISTL + one decay, with the addition that transitions for actions not taken decayed by the same rate to the prior on state transitions (i.e., 0.5):

Each trial, a transition estimate is updated with a learning rate, and probabilities are at that time normalized. For instance, if action 1 is taken and transition to state 2:

$P(s=2|a=unchosen)_{t+1} = P(s=2|a=1)_t + \gamma(0.5 - P(s=2|a=unchosen)_t)$

and

$P(s=1|a=unchosen)_{t+1} = 1 - P(s=2|a=unchosen)_{t+1}$

Second, the ISTL model include a separate free decay parameter on all unchosen actions, which is the same as defined in the Typical + two decay model.

2 Models without decay on unchosen actions

2.1 Typical model no decay

R = reward

$T = \begin{bmatrix} P(s=1|a=1) & P(s=1|a=2) \\ P(s=2|a=1) & P(s=2|a=2) \end{bmatrix}$ Transition matrix

M = one-hot vector indicating which first-stage action was taken on the last trial.

First-stage action values: $Q_{MF0_{t,a}}$ = First-stage action value predicting value at second stage.

$Q_{MF1_{t,a}}$ = First-stage action value predicting reward after second stage.

$Q_{MB_{t,a}}$ = Model-based value of action 1

Second-stage action values: $Q_{MF2_{t,s,a}}$ = Second-stage action value predicting reward.

2.1.1 Free Parameters

All alpha parameters below were drawn from Beta priors that spans the 0-1 range.

α_1 = learning rate for both updates on first-stage action values.

α_2 = learning rate for for update on second action value.

All beta parameters below were drawn from Gamma priors that spans all positive real numbers.

β_{MF0} = inverse temperature for Q_{MF0} at first stage.

β_{MF1} = inverse temperature for Q_{MF1} at first stage.

β_{MB} = inverse temperature for Q_{MB} at first stage.

β_{st} = strength of perseveration at first stage. This multiplies the M vector, which the previously enacted first-stage action.

β_{MF2} = inverse temperature for Q_{MF2} at second stage.

2.1.2 Learning computations

- Updating the transition matrix:

Each trial, a transition counter is updated. For example if state1, action1 led to state 2 once, and on the next transition, the same transition occurs, the counting matrix would be updated as follows:

$T_{counting} = \begin{bmatrix} 1+1 & 0 \\ 0 & 0 \end{bmatrix}$

T can be one of two matrices at and given trial $T_1 = \begin{bmatrix} 0.7 & 0.3 \\ 0.3 & 0.7 \end{bmatrix}$ or $T_2 = \begin{bmatrix} 0.3 & 0.7 \\ 0.7 & 0.3 \end{bmatrix}$ or $T_3 = \begin{bmatrix} 0.5 & 0.5 \\ 0.5 & 0.5 \end{bmatrix}$ at any given trial.

This is determined by the $T_{counting}$ matrix. When $T_{counting}(1,1) + T_{counting}(2,2) > T_{counting}(1,2) + T_{counting}(2,1)$, then T_1 is used. When the inequality is the converse, then T_2 is used. If they are equal, then T_3 is used.

- Updating action-values

$Q_{MF0_{t+1,a}} = Q_{MF0_{t,a}} + \alpha_1(Q_{MF2_t} - Q_{MF0_{t,a}})$

$Q_{MF1_{t+1,a}} = Q_{MF1_{t,a}} + \alpha_1(R - Q_{MF1_{t,a}})$

$Q_{MF2_{t+1,s,a}} = Q_{MF2_{t,s,a}} + \alpha_2(R - Q_{MF2_{t,s,a}})$

Model-based q-values are then computed via the Bellman equation:

$Q_{MB_{t+1}} = P(s_1|a_i) \max_{a\in\{1,2\}} Q_{MF2}(s_1, a_i) + P(s_2|a_i) \max_{a\in\{1,2\}} Q_{MF2}(s_2, a_i).$

2.1.3 Decision computations

First-stage action:

$P(a) \propto e^{(\beta_{MF0}Q_{MF0} + \beta_{MF1}Q_{MF1} + \beta_{MB}Q_{MB} + \beta_{st}M)}$

Secon-stage action:

$P(a, s_i) \propto e^{(\beta_{MF2}Q_{MF2})}$

3 Incremental State Transition Learning (ISTL) model without decay

R = reward

$T = \begin{bmatrix} P(s=1|a=1) & P(s=1|a=2) \\ P(s=2|a=1) & P(s=2|a=2) \end{bmatrix}$ transition matrix

M = one-hot vector indicating which first-stage action was taken on the last trial.

First-stage action values: $Q_{MF0_{t,a}}$ = First-stage action value predicting value at second stage.

$Q_{MF1_{t,a}}$ = First-stage action value predicting reward after second stage.

$Q_{MB_{t,a}}$ = Model-based value of action 1

Second-stage action values: $Q_{MF2_{t,s,a}}$ = Second-stage action value predicting reward.

3.0.1 Free Parameters

All alpha parameters below were drawn from Beta priors that spans the 0-1 range.

α_1 = learning rate for both updates on first-stage action values.

α_2 = learning rate for for update on second action value.

γ = learning rate for state transitions

All beta parameters below were drawn from Gamma priors that spans all positive real numbers.

β_{MF0} = inverse temperature for Q_{MF0} at first stage.

β_{MF1} = inverse temperature for Q_{MF1} at first stage.

β_{MB} = inverse temperature for Q_{MB} at first stage.

β_{st} = strength of perseveration at first stage. This multiplies the M vector, which the previously enacted first-stage action.

β_{MF2} = inverse temperature for Q_{MF2} at second stage.

3.0.2 Learning computations

- Updating the transition matrix:

$T = \begin{bmatrix} P(s=1|a=1) & P(s=1|a=2) \\ P(s=2|a=1) & P(s=2|a=2) \end{bmatrix}$

Each trial, a transition estimate is updated with a learning rate, and probabilities are at that time normalized. For instance, if action 1 is taken and transition to state 2:

$P(s=2|a=1)_{t+1} = P(s=2|a=1)_t + \gamma(1 - P(s=2|a=1)_t)$

and

$PP(s=1|a=1)_{t+1} = 1 - P(s=2|a=1)_{t+1}$

- Update action values:

$Q_{MF0_{t+1,a}} = Q_{MF0_{t,a}} + \alpha_1(Q_{MF2_t} - Q_{MF0_{t,a}})$

$Q_{MF1_{t+1,a}} = Q_{MF1_{t,a}} + \alpha_1(R - Q_{MF1_{t,a}})$

$Q_{MF2_{t+1,s,a}} = Q_{MF2_{t,s,a}} + \alpha_2(R - Q_{MF2_{t,s,a}})$

Model-based q-values are then computed via the Bellman equation:

$Q_{MB_{t+1}} = P(s_1|a_i) \max_{a\in\{1,2\}} Q_{MF2}(s_1, a_i) + P(s_2|a_i) \max_{a\in\{1,2\}} Q_{MF2}(s_2, a_i).$

3.0.3 Decision computations

First-stage action:

$P(a) \propto e^{(\beta_{MF0}Q_{MF0}+\beta_{MF1}Q_{MF1}+\beta_{MB}Q_{MB}+\beta_{st}M)}$

Secon-stage action:

$P(a, s_i) \propto e^{(\beta_{MF2}Q_{MF2})}$

α_1 = learning rate for Q_{MF0}

α_2 = learning rate for Q_{MF1} and Q_{MB}

4 ISTL + no decay + Counterfactual

Same as ISTL + no decay except that transitions for actions not taken are updated as if the not-taken action led to the state than was not experienced for the taken action. This counterfactual inference is predicated on assumption (that was told to participants and experienced in practice) that the two actions cannot lead most often to the same state.

5 ISTL + no decay + Dynamic LR

Same as ISTL + no decay except here the γ decays to 0 on each trial by the following equation:

$\gamma_t = \frac{1}{\epsilon + \mathrm{N}_{action}}$

where ϵ determine the starting learning rate, and N_{action} is a tally of how many times a given action was taken.

6 Dynamic LR + Intercept

Same asISTL + no decay + Dynamic LR except here the γ decays to a variable baseline LR, ω:

$\gamma_t = \omega + \frac{1-\omega}{\epsilon + \mathrm{N}_{action}}$

where ϵ determines time it will take to decay to baselin ω learning rate, and N_{action} is a tally of how many times a given action was taken.

7 Fixed LR

Same as 2AV + LR except here, γ is fixed across subjects.

8 Bayesian transition learning

R = reward

$T = \begin{bmatrix} P(s=1|a=1) & P(s=1|a=2) \\ P(s=2|a=1) & P(s=2|a=2) \end{bmatrix}$ transition matrix

M = one-hot vector indicating which first-stage action was taken on the last trial.

First-stage action values: $Q_{MF0_{t,a}}$ = First-stage action value predicting value at second stage.

$Q_{MF1_{t,a}}$ = First-stage action value predicting reward after second stage.

$Q_{MB_{t,a}}$ = Model-based value of action 1

Second-stage action values: $Q_{MF2_{t,s,a}}$ = Second-stage action value predicting reward.

- Transition matrices:

p_1 represents the belief that the true transition matrix is $T_1 = \begin{bmatrix} 0.7 & 0.3 \\ 0.3 & 0.7 \end{bmatrix}$

Whereas p_2 represents the belief that the true transition matrix is $T_2 = \begin{bmatrix} 0.3 & 0.7 \\ 0.7 & 0.3 \end{bmatrix}$ at any given trial.

8.0.1 Fixed parameter

The beta prior defining evidence in favor of Transition Matrix 1 was initialized with mode=0.5

8.0.2 Free Parameters

All alpha parameters below were drawn from Beta priors that spans the 0-1 range.

α_1 = learning rate for both updates on first-stage action values.

α_2 = learning rate for for update on second action value.

κ = concentration of prior over belief in either possible transition matrix.

All beta parameters below were drawn from Gamma priors that spans all positive real numbers.

β_{MF0} = inverse temperature for Q_{MF0} at first stage.

β_{MF1} = inverse temperature for Q_{MF1} at first stage.

β_{MB} = inverse temperature for Q_{MB} at first stage.

β_{st} = strength of perseveration at first stage. This multiplies the M vector, which the previously enacted first-stage action.

β_{MF2} = inverse temperature for Q_{MF2} at second stage.

8.0.3 Learning computations

- Updating the transition matrix:

Note that we use a mode of 0.5 to define the prior belief in the correct transition matrix, and a free parameter to quantify the spread of the belief distribution, which is formally known as the concentration of the prior distribution.

The mode (fixed) and concentration (free) of the beta distribution defining the prior belief in T1 and T2 was converted to E1 (evidence in favor of T1) and E2 (evidence in favor of T2) parameters describing the shape of the beta distribution by the following equations:

$E1 = \text{mode}(\kappa - 2) + 1.$

$E2 = \text{(1-mode)}(\kappa - 2) + 1.$

The posterior of the beta prior is updated analytically:

$E1 = E1 + 1$ when common transitions predicted by T1 are experienced.

and

$E2 = E2 + 1$ when common transitions predicted by T2 are experienced.

Each time model-based action values are computed, evidence for each transition matrix is derived from the mean of the beta distribution by:

$p_1 = \frac{E1}{E1+E2}$ which represents the probability that T1 is the true transition matrix.

$p_2 = 1 - p_1.$

$Q_{MB_{t+1}} = \text{(Bellman Equation for } T_1)(p_1) + \text{(Bellman Equation for } T_2)(p_2).$

- Updating action-values

$Q_{MF0_{t+1,a}} = Q_{MF0_{t,a}} + \alpha_1(Q_{MF2_t} - Q_{MF0_{t,a}})$

$Q_{MF1_{t+1,a}} = Q_{MF1_{t,a}} + \alpha_1(R - Q_{MF1_{t,a}})$

$Q_{MF2_{t+1,s,a}} = Q_{MF2_{t,s,a}} + \alpha_2(R - Q_{MF2_{t,s,a}})$

Model-based q-values are then computed via the Bellman equation:

$Q_{MB_{t+1}} = P(s_1|a_i) \max_{a \in \{1,2\}} Q_{MF2}(s_1, a_i) + P(s_2|a_i) \max_{a \in \{1,2\}} Q_{MF2}(s_2, a_i).$

8.0.4 Decision computations

First-stage action:

$P(a) \propto e^{(\beta_{MF0}Q_{MF0} + \beta_{MF1}Q_{MF1} + \beta_{MB}Q_{MB} + \beta_{st}M)}$

Secon-stage action:

$P(a, s_i) \propto e^{(\beta_{MF2}Q_{MF2})}$

9 MB

Here, first-stage actions are only influences by model-based planning and a perseveration parameter.

$R =$ reward

$T = \begin{bmatrix} P(s=1|a=1) & P(s=1|a=2) \\ P(s=2|a=1) & P(s=2|a=2) \end{bmatrix}$ transition matrix

$M =$ one-hot vector indicating which first-stage action was taken on the last trial.

First-stage action values: $Q_{MF0_{t,a}} =$ First-stage action value predicting value at second stage.

$Q_{MF1_{t,a}} =$ First-stage action value predicting reward after second stage.

$Q_{MB_{t,a}} =$ Model-based value of action 1

Second-stage action values: $Q_{MF2_{t,s,a}} =$ Second-stage action value predicting reward.

9.0.1 Free Parameters

All alpha parameters below were drawn from Beta priors that spans the 0-1 range.

$\alpha_1 =$ learning rate for both updates on first-stage action values.

$\alpha_2 =$ learning rate for for update on second action value.

$\gamma =$ learning rate for state transitions

All beta parameters below were drawn from Gamma priors that spans all positive real numbers.

$\beta_{MB} =$ inverse temperature for Q_{MB} at first stage.

$\beta_{st} =$ strength of perseveration at first stage. This multiplies the M vector, which retains which action was taken most recently.

$\beta_{MF2} =$ inverse temperature for Q_{MF2} at second stage.

9.0.2 Learning computations

- Updating the transition matrix:

$T = \begin{bmatrix} P(s=1|a=1) & P(s=1|a=2) \\ P(s=2|a=1) & P(s=2|a=2) \end{bmatrix}$

Each trial, a transition estimate is updated with a learning rate, and probabilities are at that time normalized. For instance, if action 1 is taken and transition to state 2:

$P(s=2|a=1)_{t+1} = P(a_1, s_2)_t + \gamma(1 - P(s=2|a=1)_t)$

and

$P(s=1|a=1)_{t+1} = 1 - P(s=2|a=1)_{t+1}$

- Updating action-values

$Q_{MF0_{t+1,a}} = Q_{MF0_{t,a}} + \alpha_1(Q_{MF2_t} - Q_{MF0_{t,a}})$

$Q_{MF1_{t+1,a}} = Q_{MF1_{t,a}} + \alpha_1(R - Q_{MF1_{t,a}})$

$Q_{MF2_{t+1,s,a}} = Q_{MF2_{t,s,a}} + \alpha_2(R - Q_{MF2_{t,s,a}})$

Model-based q-values are then computed via the Bellman equation:

$Q_{MB_{t+1}} = P(s_1|a_i) \max_{a \in \{1,2\}} Q_{MF2}(s_1, a_i) + P(s_2|a_i) \max_{a \in \{1,2\}} Q_{MF2}(s_2, a_i).$

9.0.3 Decision computations

First-stage action:

$P(a) \propto e^{(\beta_{MB} Q_{MB} + \beta_{st} M)}$

Secon-stage action:

$P(a, s_i) \propto e^{(\beta_{MF2} Q_{MF2})}$

10 1AV Model

R = reward

$T = \begin{bmatrix} P(s=1|a=1) & P(s=1|a=2) \\ P(s=2|a=1) & P(s=2|a=2) \end{bmatrix}$ transition matrix

First-stage action values: $Q_{MF1_{t,a}}$ = First-stage action value.

$Q_{MB_{t,a}}$ = Model-based value of action 1

M = one-hot vector indicating which first-stage action was taken on the last trial.

Second-stage action values: $Q_{MF2_{t,s,a}}$ = Second-stage action value predicting reward.

10.0.1 Free Parameters

All parameters below were drawn from Beta priors that spans the 0-1 range.

α_1 = learning rate for both updates on first-stage action values.

α_2 = learning rate for for update on second action value.

λ = eligibility trace

ω = weight on model-based control

All beta parameters below were drawn from Gamma priors that spans all positive real numbers.

β_1 = inverse temperature for Q_{MF0} at first stage.

β_2 = inverse temperature for Q_{MB} at first stage.

st = perseveration parameter

10.0.2 Learning computations

- Updating the transition matrix:

Each trial, a transition counter is updated. For example if state1, action1 led to state 2 once, and on the next transition, the same transition occurs, the counting matrix would be updated as follows:

$T_{counting} = \begin{bmatrix} 1+1 & 0 \\ 0 & 0 \end{bmatrix}$

T can be one of two matrices at and given trial $T_1 = \begin{bmatrix} 0.7 & 0.3 \\ 0.3 & 0.7 \end{bmatrix}$ or $T_2 = \begin{bmatrix} 0.3 & 0.7 \\ 0.7 & 0.3 \end{bmatrix}$ or $T_3 = \begin{bmatrix} 0.5 & 0.5 \\ 0.5 & 0.5 \end{bmatrix}$ at any given trial.

This is determined by the $T_{counting}$ matrix. When $T_{counting}(1,1)+T_{counting}(2,2) > T_{counting}(1,2)+T_{counting}(2,1)$, then T_1 is used. When the inequality is the converse, then T_2 is used. If they are equal, then T_3 is used.

- Updating action-values

$Q_{MF1_{t+1,a}} = Q_{MF1_{t,a}} + \alpha_1(Q_{MF2_t} - Q_{MF_{t,a}})$

$Q_{MF1_{t+2,a}} = Q_{MF1_{t+1,a}} + \alpha_1(R - Q_{MF1_{t+1,a}})$

$Q_{MF2_{t+1,s,a}} = Q_{MF2_{t,s,a}} + \alpha_2(R - Q_{MF2_{t,s,a}})$

Model-based q-values are then computed via the Bellman equation:

$Q_{MB_{t+1}} = P(s_1|a_i) \max_{a\in\{1,2\}} Q_{MF2}(s_1, a_i) + P(s_2|a_i) \max_{a\in\{1,2\}} Q_{MF2}(s_2, a_i)$.

Q values for 1-stage actions are integrated in the following way:

$Q_{integrated} = \omega(Q_{MB}) + (1-\omega)(Q_{MF1})$

10.0.3 Decision computations

First-stage action:

$P(a) \propto e^{\beta_1[Q_{integrated}+st(M)]}$

Secon-stage action:

$P(a, s_i) \propto e^{\beta_2 Q_{MF2}}$

11 1AV+ LR model

Same as Daw model except state transition estimates are learned in the same way as 2AV + LR.

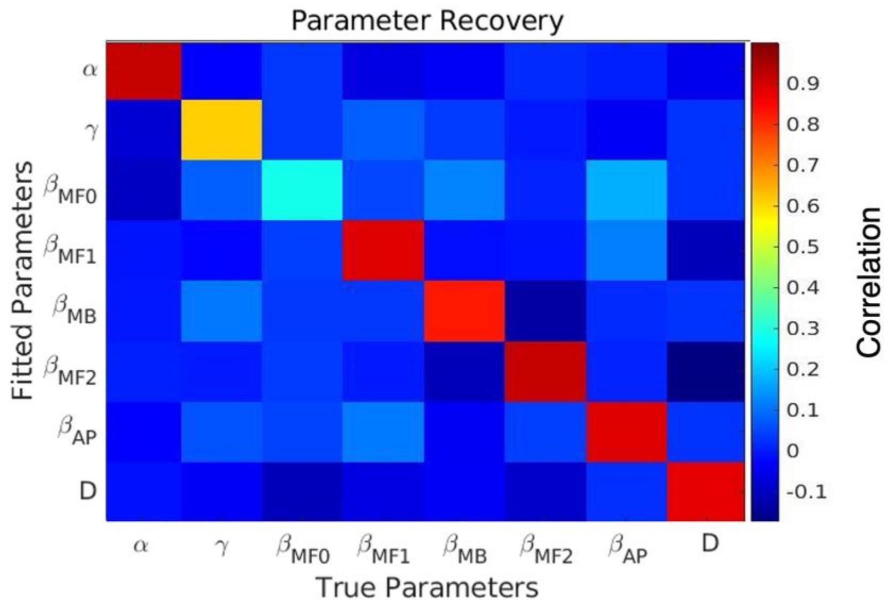

Figure S4: Parameter Recovery. The diagram comprises data simulated from the winning model (ISTL) and best-fit group hyperparameters for the subsample of subjects (MB > 2.5). The simulation was contained 400 generated agents over 200 trials of the two-step task. The group hyperparameters were derived through fitting the model, and thus, parameter recovery reflect the empirical range of parameter values. The plot comprises the full set of correlations between fitted and true parameters in the simulation and subsequent model fitting. The vertical axis in the heatmap with "F" represent the fitted parameters, and the horizontal axis represent the ground-truth parameters that generated the data. The following are the group priors we generated the data from. Importantly, next to each parameter in parentheses denotes the abbreviations used in the heatmap for each of these parameters:

Learning rates:
Learning Rate (α) ~ beta(1.76,0.57)
Learning Rate state transitions(γ) ~ beta(0.50,3.60).
Decay (d) ~ beta(1.50,3.50)
Softmax beta weights 1st action:
Model based beta (β_{MB}) ~ gamma(0.5,10) lower bounded at 2.5
Model free beta TD0 (β_{MF0}) ~ gamma(0.6,0.2)
Model free beta TD1(β_{MF1}) ~ gamma (1.02,1.32)**,**
Action perseveration beta (β_{AP}) ~ normal (0.84,0.73).
Softmax beta weight 2nd action:
Model free beta 2nd stage (β_{MF2}) ~ gamma (3.03,0.8).

Note S1. Model-fitting and model recovery

Simulating data from both the winning model ('ISTL'; incremental state transition learning rate) and the second-best fitting model ('Typical'; typical model used in prior studies that use a counting rule for state transition learning), we recovered the generative model 10 out of 10 times, using group-fitted population parameters. In other words, when generating the data from 'ISTL', we found that 'ISTL' explained the resultant simulated data far better than the next-best-fitting model, 'Typical'. We then simulated data as if 'Typical' model was in fact true, and also found that the resultant data was explained better by 'Typical' than did the 'ISTL' model. Specifically, the mean difference in iBIC when generating data with the winning model, was 332.99 in favor of 'ISTL' when 'ISTL' generated the data, and was 196.41 in favor of 'Typical' when generating the data with 'Typical'.

The priors used to generate data for 'ISTL' are written above in Figure S5.

The priors for 'Typical' are:

Learning Rate (α) ~ beta(1.76,0.57)
Learning Rate state transitions(γ) ~ beta(0.50,3.60).
Decay (d) ~ beta(1.50,3.50)
Softmax beta weights 1st action:
Model based beta (β_{MB}) ~ gamma(0.5,10) lower bounded at 2.5
Model free beta TD0 (β_{MF0}) ~ gamma(0.6,0.2)
Model free beta TD1(β_{MF1}) ~ gamma (1.02,1.32)**,**
Action perseveration beta (β_{AP}) ~ normal (0.84,0.73).
Softmax beta weight 2nd action:
Model free beta 2nd stage (β_{MF2}) ~ gamma (3.03,0.8).

The hierarchical model-fitting procedure we describe in text was used to fit models both to empirical and simulated data. All parameters drawn from beta distributions started with uniform priors over the [0,1] range of possible values, and all other parameters drawn from gamma distributions started with priors that favored small values (scale=1, rate=1) over the [0,∞] range.

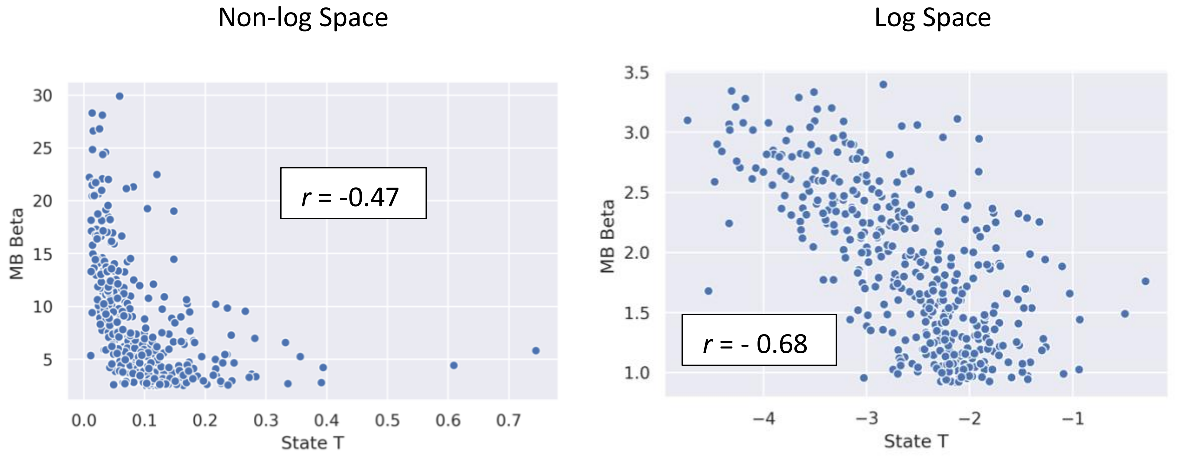

Figure S5. Log-transforming accounts for more shared variance between LR and MB-Beta. We demonstrate that log-transforming state transition learning rate and model-based beta computational parameters increases their shared variance, evidenced by the significantly increased correlation between the two variables when doing so. Importantly, we sought to maximally account for such shared variance in regressions reported in Results.
